# Multi-Perspective Spatio-Temporal Feature Fusion Model for Urban Traffic Flow Prediction

**DOI:** 10.3390/s26154744

**Published:** 2026-07-26

**Authors:** Avazjon Marakhimov, Rustem Jalelov, Jabbar Kudaybergenov, Zahriddin Muminov, Kabul Khudaybergenov, Shukhrat Tajibaev

**Affiliations:** 1 Department of Information Processing and Management Systems, Tashkent State Technical University, Tashkent 100174, Uzbekistan; a.marakhimov@tdtu.uz (A.M.); r.m.jalelov@gmail.com (R.J.); 2 Department of Software Engineering, Nukus State Technical University, Nukus 100147, Uzbekistan; kjabbarbergen@umail.uz; 3 Department of Applied Mathematics, Tashkent State University of Economics, Tashkent 100066, Uzbekistan; zimuminov@gmail.com; 4 Department of Applied Informatics, Kimyo International University in Tashkent, Tashkent 100121, Uzbekistan; 5 Department of Exact Sciences, Mamun University, Urganch 220900, Uzbekistan; tajibaevsx@mamunedu.uz

**Keywords:** traffic flow prediction, spatio-temporal correlation, temporal fusion, self-attention mechanism, graph neural networks, convolutional neural networks

## Abstract

Urban traffic flow is difficult to forecast accurately because its evolution is non-linear and governed by dependencies that operate over different spatial and temporal ranges. This paper introduces the Multi-Perspective Spatio-Temporal Feature Fusion Model (MPSTFFM) to describe these dependencies through complementary views. The temporal signal is separated into a slowly varying trend and a residual fluctuation, while the spatial structure is represented by four graphs: first-order adjacency, second-order in-degree, second-order out-degree, and a data-adaptive graph. These graphs respectively encode physical road connectivity, common inflow sources, common outflow destinations, and latent spatial associations. Whereas the first three are constructed from the known network topology, the adaptive graph is learned together with the prediction model and can therefore identify correlations not expressed by physical links. Within each spatio-temporal view, self-attention captures dependencies over long ranges, and convolutional operations extract local patterns. The features learned from all views are subsequently fused into a high-dimensional representation used to predict future flow. Experiments on real-world datasets compare MPSTFFM with twelve methods published during the preceding five years. On these benchmarks MPSTFFM outperforms every baseline, lowering the average MAE, RMSE, and MAPE across the four datasets by 13.04%, 5.28%, and 9.59%, respectively, relative to the best baseline on each one.

## 1. Introduction

Rapid urban growth has intensified the mismatch between road capacity and travel demand, making congestion a routine condition in many large cities [1]. The consequences extend beyond additional travel time and include greater fuel consumption, poorer air quality, economic losses, and restrictions on sustainable urban development. At the same time, increasingly dense sensor networks have created large repositories of traffic measurements that can support accurate short-term forecasting [2]. Such forecasts help travelers select more efficient routes and allow traffic authorities to anticipate bottlenecks, adjust signal control, and plan infrastructure more effectively.

The practical value of forecasting depends on both accuracy and timeliness. A prediction delivered before congestion develops can support preventive control, whereas an estimate produced after conditions have changed has limited operational value. Network-level forecasts are also needed because an intervention at one junction may redistribute traffic to several neighboring roads. Consequently, traffic management requires models that can process large sensor networks efficiently while preserving the interactions through which local changes spread across an urban area.

The forecasting problem remains challenging because road traffic does not evolve as an isolated linear sequence. Flow on each segment responds non-linearly to changes in demand and external disturbances, while also being influenced by conditions on surrounding roads. Moreover, measurements exhibit both temporal and spatial autocorrelation, and the relevant dependency range changes with the time scale and geographic extent under consideration [2]. A useful model must therefore account for several interacting forms of dependence rather than represent space or time in isolation.

These interactions are heterogeneous. Regular commuting demand produces smooth and repeatable temporal structure, whereas incidents, weather changes, or short-lived surges generate rapid deviations from that structure. Spatial relationships are equally varied: directly connected intersections exchange vehicles physically, locations with common upstream sources may experience related inflow, and distant areas with similar functions can follow comparable daily profiles despite having no connecting edge. Treating all of these relationships as a single homogeneous dependency can obscure the mechanisms that are most relevant to a particular prediction.

Early deep-learning approaches mapped traffic observations onto regular grids and processed them as images with convolutional neural networks (CNNs) [3,4,5]. This formulation can extract local patterns, but it neither models long temporal ranges naturally nor preserves the irregular connectivity of actual road systems. Recurrent neural networks (RNNs) [1,2], including gated recurrent units and long short-term memory networks [6,7,8,9,10,11,12,13], subsequently became common because their hidden states transfer information across successive observations. Although this improves temporal modeling, recurrent architectures may be difficult to train, prone to overfitting, and unable to describe road-network structure adequately.

Graph neural networks (GNNs) address the spatial limitation by operating directly on the network topology. When combined with recurrent, convolutional, or Transformer modules, they propagate information between connected locations and learn joint spatio-temporal representations [3,7,9,10,14,15,16,17,18,19,20,21,22,23,24]. Data-adaptive graphs have also been introduced to represent correlations that change with observed flow and are not visible in the physical topology. Nevertheless, most existing formulations give limited explicit attention to travel direction from upstream to downstream intersections. They also tend to encode traffic through a single spatial or temporal representation rather than systematically combining several complementary views.

This limitation is important for directed road systems. Symmetrizing a graph simplifies convolution, but it makes a road entering an intersection indistinguishable from a road leaving it. A model may then know that two locations are related without knowing whether their relationship reflects incoming or outgoing traffic. Adaptive adjacency can discover additional associations, yet a learned graph alone does not guarantee that physically meaningful directional structure will be retained. A representation that combines fixed topology, directional second-order relations, and data-driven dependence can preserve these different sources of spatial information instead of forcing one graph to approximate all of them.

To address these limitations, we develop the Multi-Perspective Spatio-Temporal Feature Fusion Model (MPSTFFM). Its temporal decomposition separates the observed series into a stable trend and a rapidly varying residual. Its spatial decomposition represents the network through adjacency, in-degree, out-degree, and adaptive graphs, thereby combining fixed directional structure with relationships learned from data. The topology-derived graphs retain known road information, while the adaptive graph connects locations with similar behavior even when no direct road link exists. MPSTFFM learns local and long-range dependencies independently within the eight combinations of temporal and spatial views, then fuses their outputs into a common high-dimensional representation. The main contributions are as follows:

This formulation treats decomposition as a representation strategy rather than a preprocessing step that discards information. The original temporal signal is exactly divided between trend and residual, and the four graphs offer alternative descriptions of the same network instead of competing estimates of one adjacency. All views remain available to the learning system, which determines their relative value through the extraction and fusion parameters. The approach is therefore intended to increase representational diversity while retaining the observed traffic content and the known physical structure.

- We formulate a feature-fusion architecture that learns traffic representations from several spatial and temporal perspectives in parallel and combines them to improve prediction over a single-view formulation.
- The temporal decomposition isolates persistent supply–demand behavior from short-lived variation by separating trend and fluctuation components, which improves robustness to noisy observations and changing traffic conditions.
- The spatial representation combines direct connectivity, common-upstream similarity, common-downstream similarity, and learned latent association through four distinct graphs. The second-order graphs recover directional information that is usually lost when a road network is symmetrized, while the adaptive graph identifies dependencies between topologically distant intersections with similar behavior.
- We implement the complete MPSTFFM forecasting procedure and evaluate it on public datasets, where it consistently outperforms twelve recent baseline methods.

## 2. Related Work

### 2.1. Traffic Flow Prediction

Traffic flow forecasting infers future network conditions from patterns embedded in past observations. Before deep learning became dominant, temporal variation was modeled primarily with statistical techniques [25,26] and conventional machine learning methods [27,28]. These approaches provided important foundations, but their treatment of spatial context was generally weak, and their limited non-linear capacity restricted performance on complex traffic dynamics.

Classical models remain attractive when data are limited because their parameters are often easier to interpret and estimate. Their assumptions, however, become restrictive in large networks where relationships vary by location and time. A separate model for every sensor does not naturally share information across the network, while a single global model may overlook local differences. The growth of high-frequency traffic archives therefore encouraged methods that learn spatial and temporal features jointly rather than specifying them manually.

Deep neural networks expanded the range of relationships that could be learned directly from data. RNN-based sequence-to-sequence models [1,2], particularly GRU [6,7,8,9] and LSTM variants [10,11,12,13], became widely used for non-linear temporal modeling. CNNs were applied to grid-form traffic maps to extract local spatial features [4,5], and several hybrid models combined recurrent and convolutional modules to process space and time jointly [6,7,29]. The regular-grid assumption, however, does not match the non-Euclidean form of a road network and cannot faithfully preserve irregular topological relationships.

By operating on graph-structured inputs, GNNs avoid this mismatch and propagate features through arbitrary network links. Their integration with RNNs, attention mechanisms, and CNNs has substantially improved joint spatio-temporal modeling [7,8,10,14,15,16,17,30,31,32,33,34,35]. Remaining concerns include sensitivity to noisy measurements, difficulty combining heterogeneous information, and insufficient temporal reach when relevant dependencies extend beyond the effective receptive field. These limitations leave room for architectures that are both more expressive and more robust.

Related architectures have also been proposed for other networks and data sources. Alsehaimi et al. [36] combine self-attention with several graph views to track changing spatio-temporal patterns. Jeon and Jeong [37] pair spatio-temporal graph convolution with CNN layers to predict short-term speed from probe-vehicle data in a South Korean city, and Singh et al. [38] stack multi-layer graph convolution with sequential convolutional blocks in ISTGCN. Karthika and Uma Maheswari [39] instead treat graph selection as a multi-objective problem, combining adaptive sampling with Pareto-based optimization to balance competing accuracy criteria. Anitha et al. [40] embed traffic graphs in hyperbolic space and add a quantum graph layer together with neural ordinary differential equations to capture hierarchical network structure, while Mundada et al. [41] apply variational mode decomposition ahead of a federated graph-attention and LSTM pipeline so that intrinsic traffic components can be learned without centralizing sensor data. Across these studies, the graphs and attention modules discussed above are applied to a wide range of networks and settings, though the spatial representation is typically still a single view.

Repeated graph propagation can also blur distinctions between node representations when many layers are stacked. Increasing depth enlarges the receptive field, but it raises optimization cost and does not ensure that distant information remains useful after several aggregation steps. Attention layers avoid strictly hop-by-hop propagation, although they may weaken the local structural bias supplied by the graph. The two mechanisms are therefore complementary: graph convolution is naturally suited to neighborhood structure, while attention provides direct access to remote locations and observations.

Large language models (LLMs) have recently been explored as an alternative forecasting paradigm because of their strong representation and reasoning abilities. Li et al. [42] supplied structured spatio-temporal dependencies to a GPT model and reported improvements in accuracy and transfer across scenarios. Liu et al. [43] and Xu et al. [44] incorporated spatial position and global temporal patterns into LLM inputs, using frozen pre-trained backbones to avoid complete retraining. Guo et al. [45] converted traffic measurements and external context, including points of interest, weather, weekday information, and holidays, into natural-language descriptions so that an LLM could reason over their interactions. Rong et al. [46] combined LLMs with edge computing to lower the inference delay of a generative spatio-temporal framework for large 6G transportation systems. Despite their promise, LLM-based methods remain computationally expensive, slow at inference, difficult to interpret, and not naturally suited to precise numerical relationships across space and time.

### 2.2. Spatio-Temporal Relationship Models

GCNs are frequently paired with temporal modules so that spatial propagation and sequence dynamics can be learned within the same forecasting system. Jin et al. [14] integrated automatically dilated graph convolution, Gated Linear Units (GLU), and temporal convolutional networks (TCN) in a synchronous architecture whose receptive field adapts to dependencies of different ranges. The two-stage model of Huang et al. [9] first combines graph attention with GCN-based adjacency modeling and then uses GLU to merge long- and short-range features. Zhong et al. [10] formed an ST-GCN by coupling GCN and LSTM layers to learn extended dependencies between intersections. Huang et al. [17] instead generated time-dependent graphs for individual periods and processed them with a GCN–GRU network to obtain multi-scale patterns. T-GCN [8] follows a more direct sequence: a GCN embeds road topology into node representations, after which a GRU models their evolution over time.

Other designs place the graph operator inside the recurrent unit rather than connecting separate spatial and temporal modules. DCRNN [7], for example, replaces standard GRU matrix operations with diffusion graph convolution, so every hidden-state transition includes information from nearby graph nodes. SpAttRNN [11] similarly introduces learned spatial weighting by substituting a graph attention network for the LSTM memory unit. Zhao et al. [15] construct graphs from current observations, replace the GRU gates with GCN layers, and recursively propagate several spatial relationships through the recurrent process. Loosely coupled modules are generally easier to implement and modify, but they may not fully capture interactions between space and time. Embedding graph computation within recurrence expresses these interactions more directly, although at the cost of greater training complexity, computation, and constraints on input organization.

The choice between these designs is therefore not purely architectural. It determines when spatial information enters temporal processing and whether the same graph relation is reused at every recurrent step. A fixed integration scheme may be effective for stable traffic patterns but less suitable when the importance of spatial relationships changes across temporal components. Processing several perspectives independently offers another possibility: each branch can specialize before the information is merged, reducing the pressure on one recurrent or convolutional pathway to encode every dependency simultaneously.

A second limitation arises when adjacency is defined exclusively by physical roads. Fixed topology cannot describe functional similarity between remote locations that exhibit related traffic patterns. Dynamic graph methods instead infer edges from observations [47,48], producing relationships based on traffic similarity or semantic correspondence. This allows geographically distant but temporally correlated nodes to exchange information and increases the effective dependency range. The benefit comes with additional graph-learning cost, and a learned graph may not respond quickly enough when traffic changes abruptly or anomalously.

Transformers offer another route to long-range modeling and have become prominent in traffic forecasting after their success in language and vision. Xiao et al. [19] combined graph convolution with self-attention in a single spatio-temporal module. GMAN [49] extended multi-head attention to both space and time through an encoder-decoder that learns changing relationships between nodes. TFMGCAM [34] joined a Transformer encoder with GCN layers to represent global sequences and local graph structure simultaneously. Trafformer [18] used a Transformer-derived correlation matrix as a shared encoder input, aligning spatial and temporal feature spaces. PDFormer [20] incorporated traffic propagation delay into attention weights, while ST-CGA [50] stacked Transformer attention and multi-head graph attention to combine detailed temporal patterns with wider spatial context. DEC-Former [35] separated forecasting into a Fourier temporal-convolution branch and a Transformer spatial-attention branch, then merged them with Gaussian attention. Pairwise attention gives these methods a global receptive field and permits the inclusion of contextual variables such as weather and calendar information. However, their computational cost grows quadratically, their performance can deteriorate under distribution shift, and effective training often requires substantial data.

Similar architectures are also being applied beyond benchmark accuracy, in policy and infrastructure settings. Bouchemoukha et al. [51] fuse gated temporal convolution with graph-based spatial attention in a spatial-temporal graph gated transformer, and Mandlik et al. [52] use a multi-head-attention spatio-temporal graph network for freeway speed prediction on a North American corridor. Edalatpanah and Pourqasem [53] take a different route with DSTGN-ExpertNet, routing traffic patterns through a mixture-of-experts gate so that specialized temporal sub-networks share a common graph-based spatial encoding. At a broader level, Capone et al. [54] survey spatio-temporal prediction with graph neural networks across application domains, and Albalooshi [55] and Abduljabbar et al. [56] evaluate deep-learning and classical machine-learning pipelines for traffic flow prediction in smart-city and sustainable-transportation contexts. Graph-based spatio-temporal modeling is therefore not confined to one dataset or region; it is being adapted to a range of networks, objectives, and deployment constraints.

Traffic applications also require attention to preserve numerical structure rather than only semantic similarity. Two nodes can have similar normalized patterns but very different absolute flow levels, and an unconstrained attention score may emphasize their resemblance without respecting network feasibility. Graph-based local operators provide a useful counterbalance because their aggregation follows explicit relations. This motivates hybrid designs in which attention and graph convolution are assigned distinct responsibilities instead of being treated as interchangeable feature extractors.

Several recent approaches depart from these standard architectural combinations. STD-MAE [57] adopts self-supervised masked reconstruction: portions of the series are hidden across space and time, and the encoder learns broad context by recovering the missing observations before task-specific fine-tuning. STGODE [21] builds separate geographic and semantic spatial representations, combines them with TCN features, and models long-range evolution through a continuous-time graph network derived from ordinary differential equations. STG-NCDE [22] uses neural controlled differential equations, which are well suited to irregular sampling, to describe temporal development and replaces conventional graph convolution with a controlled differential formulation for spatial propagation. Although differential-equation models have appealing theoretical properties, they may be difficult to adapt across varied datasets and can struggle with rapidly changing, strongly non-linear traffic behavior.

To position the proposed framework against representative prior models, three widely used architectures are considered directly. Graph WaveNet pioneered the self-adaptive adjacency matrix, learning node embeddings whose product supplements a predefined graph inside a single dilated-convolution pathway; however, the learned and predefined graphs are merged within one propagation stack, directional information is lost once the topology is symmetrized, and the raw series is processed as a single temporal signal. ASTGNN captures the dynamics and heterogeneity of traffic through trend-aware temporal attention and dynamic graph convolution, but both mechanisms act on one input view and one spatial relation, so a single pathway has to encode every form of dependence at once. PDFormer comes closest to a multi-relational design: its attention heads are masked by a geographic neighborhood and a semantic (similarity-based) neighborhood, and propagation delay is injected into the attention weights. Even so, its semantic relation is fixed by a preprocessing rule (historical-pattern similarity) rather than learned against the forecasting objective, it has no counterpart to the second-order in-degree and out-degree graphs that restore directional source-sink structure, and its spatial masks share a single attention module instead of being processed by independently specialized branches.

MPSTFFM differs from these designs in three respects. The input itself is decomposed, with the temporal signal split exactly into trend and fluctuation components and the network described by four graphs whose edges rest on different evidence: direct connectivity, shared upstream supply, shared downstream demand, and a latent affinity learned jointly with the forecasting task. The eight resulting perspectives are then processed by parallel branches with unshared parameters, so each representation can specialize before anything is merged—in Graph WaveNet, ASTGNN, and PDFormer, by contrast, heterogeneous dependencies compete for capacity within a single pathway. Integration itself happens late, through learnable per-perspective weights followed by concatenation, which lets the model rebalance the spatial and temporal mechanisms for each dataset rather than fixing their relative influence in the architecture. Section 6.5 quantifies the contribution of this organization through the w/o MPST and w/o AG ablations.

## 3. Preliminaries

### 3.1. Basic Concepts

An urban road system is represented as the weighted directed graph G=(V,E,A). Here, $V=\{v_{1},v_{2},\ldots ,v_{N}\}$ is the set of monitored intersections, $E=\{e_{1},e_{2},\ldots ,e_{M}\}$ is the set of connecting road segments, and *A* denotes the adjacency weight matrix. The value $A_{ij}=1$ means that *E* contains the directed edge $e=\langle v_{i},v_{j}\rangle$ and vehicles may travel from $v_{i}$ to $v_{j}$; otherwise, $A_{ij}=0$. The upstream neighborhood of intersection $v_{i}$ is written as $up\left(v_{i}\right)=\left\{v_{j}|\exists e=\langle v_{j},v_{i}\rangle ,e\in E\right\}$.

Vehicle movement follows the directions permitted by these edges, so travel from $v_{i}$ to $v_{j}$ occurs through $e=\langle v_{i},v_{j}\rangle$. Counts collected at a fixed sampling interval form a continuous sequence of network-wide traffic observations. For forecasting at time *t*, the preceding *S* observations are organized as $X=\{X^{t-S+1},X^{t-S+2},\ldots ,X^{t}\}$. Each $X^{t}\in R^{N\times C}$ contains the measurements for all *N* intersections at that time, and *C* denotes the number of feature channels. The experiments use C=1, corresponding to a single traffic-flow count per intersection.

### 3.2. Problem Definition

Given the graph *G* and the historical tensor $X\in R^{N\times C\times S}$ covering *S* successive observations, the objective is to estimate traffic flow during the following *H* steps. Rather than supplying the complete tensor through a single representation, the proposed method separates its temporal and spatial properties into complementary views. MPSTFFM then learns the mapping ψ(·) in Equation (1), which converts the history *X* and network *G* into the future sequence *Y*.

(1) $$\left[X^{t-S+1},X^{t-S+2},\ldots ,X^{t}:G\right]\overset{\psi (\cdot )}{\underset{\left\{\left(X_{i},G_{j}\right)|X_{i}\in X,G_{j}\in G\right\}}{\to }}\left[Y^{t+1},Y^{t+2},\ldots ,Y^{t+H}\right].$$

In Equation (1), X and G denote the temporal and spatial sets generated by decomposition. Each pair $\left(X_{i},G_{j}\right)$ describes the same traffic history from a different perspective. Their learned features are fused into a higher-dimensional embedding that contains information unavailable to an individual view and is therefore better suited to prediction.

## 4. Multi-Perspective Spatio-Temporal Feature Fusion Model

### 4.1. Model Framework

**Figure 1:**
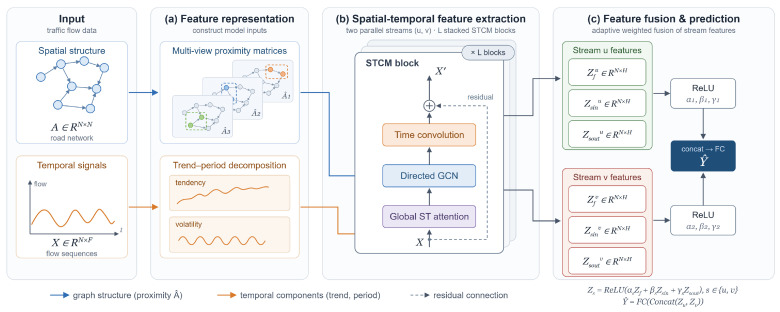
Framework diagram of the principle of MPSTFFM.

No single representation fully describes the range of dependencies present in urban traffic. MPSTFFM therefore organizes forecasting into the three-stage architecture illustrated in Figure 1. First, the input is decomposed into several temporal and spatial views. Second, a dedicated branch extracts correlations from each view. Third, the branch outputs are combined into one high-dimensional traffic representation.

Starting from $X\in R^{N\times C\times S}$ and G=(V,E,A), the decomposition in Section 4.2 generates the temporal set $\left\{X_{u},X_{v}\right\}$ and the graph set $\left\{G_{f},G_{i},G_{o},G_{a}\right\}$. Their Cartesian product forms eight input pairs (X,G). Each pair is processed independently: self-attention identifies global dependencies over long spatial and temporal ranges, whereas graph convolution aggregates information from local neighborhoods. The two temporal components distinguish persistent behavior from short-term variation, and the four graphs control the spatial relation used for propagation. Thus, every branch observes the same underlying period but emphasizes a different combination of temporal behavior and network structure. Keeping the branches separate until fusion prevents strong patterns in one representation from suppressing weaker information available in another. Learnable weights are finally applied to the eight outputs, which are concatenated into a unified description of the current traffic state.

The branch structure also separates feature discovery from feature selection. During extraction, each branch can learn parameters appropriate to its own input distribution; the trend branches need not process the rapid variations that dominate the residual, and graph-specific branches do not have to reconcile incompatible adjacency patterns inside a single convolution. During fusion, the learned weights determine how strongly the branch outputs contribute to the prediction. This organization retains specialized information until the model has produced comparable high-level representations, at which point integration is less likely to erase view-specific characteristics.

The case for multi-perspective fusion rests on the heterogeneity of the underlying dependencies. Physical vehicle transfer, shared upstream supply, shared downstream demand, and functional similarity between remote locations are distinct generative mechanisms with different statistical signatures, and the persistent trend and transient fluctuation of the same series follow different dynamics as well. A single-view model has to approximate the superposition of all these mechanisms with one set of filters, which creates two risks: dominant patterns—typically the smooth trend on the first-order graph—absorb most of the model capacity, while weaker but complementary signals, such as directional second-order relations, residual variation, or latent affinity, are attenuated before they can contribute. Decomposing the input into views with more homogeneous behavior, extracting features from them in parallel, and postponing integration until comparable high-level representations exist turns this superposition problem into a weighting problem, which the fusion parameters α then solve directly from the data.

### 4.2. Spatio-Temporal Feature Decomposition

#### 4.2.1. Temporal Feature Decomposition

We separate persistent traffic behavior from short-lived variation by filtering $X\in R^{N\times C\times S}$ with a centered moving average of width *w*. The resulting trend tensor $X_{u}\in R^{N\times C\times S}$ is defined in Equation (2).

(2) $$X_{u,i}^{t}=\frac{\sum_{k=t-\lfloor w/2\rfloor }^{t+\lfloor w/2\rfloor }X_{i}^{k}}{w}.$$

Here, $X_{i}^{k}$ is the observed count at $v_{i}$ and time *k*, while $X_{u,i}^{t}$ is its average over the *w*-step interval centered on *t*. Values outside the observed sequence are padded with zero. Subtracting the trend from the original tensor gives $X_{v}=X-X_{u}\in R^{N\times C\times S}$. This residual fluctuates around zero and records departures from the prevailing traffic pattern.

**Figure 2:**
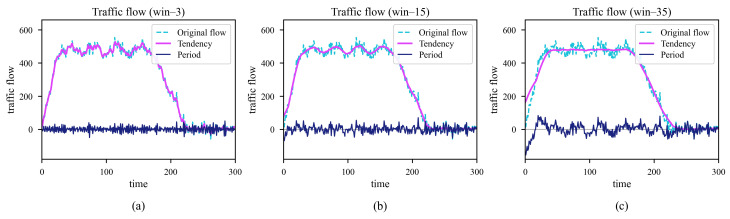
Decomposition results of temporal features of traffic flow under different sliding windows. In each subfigure, the raw traffic flow series is shown together with the extracted trend component $X_{u}$ and the residual fluctuation component $X_{v}=X-X_{u}$: (**a**) sliding window w=3; (**b**) sliding window w=15; (**c**) sliding window w=35.

The effect of *w* is illustrated in Figure 2. Regardless of the selected window, the trend follows the overall shape of the raw series while removing much of its rapid variation. It consequently reflects the more stable supply–demand structure of traffic. The residual contains the irregular changes not explained by this smooth component and varies quickly around zero. Forecasting the residual alone is more difficult, but its magnitude is comparatively small, so a large relative error need not correspond to a large absolute deviation. Decomposition therefore replaces one heterogeneous series with two components having more uniform behavior and distinct predictive roles. Adjusting *w* controls how much variation is assigned to each component.

This division does not assume that the residual is meaningless noise. Sudden demand changes and short-lived disturbances may be highly informative for near-term forecasting even when they are difficult to predict over a long horizon. Conversely, the trend provides a stable reference around which these changes occur. Learning the two components in separate branches allows the network to use different filters for their different statistical behavior, while their later fusion reconstructs a representation of the complete traffic signal.

A centered moving average is used here instead of a trailing (causal) one because a trailing window of width *w* introduces a phase delay of roughly w/2 steps: the estimated trend lags behind the true slow component, and this lag reappears with opposite sign in the residual, contaminating the fluctuation branch with displaced trend content. Centering removes this distortion, and it is legitimate here because the decomposition represents an already-observed window rather than acting as an online filter—all *S* input steps lie in the past at prediction time, so centering does not violate causality. At the boundaries of the window, where fewer than *w* observations are available, zero padding is used instead. The resulting attenuation of the trend near the edges is deterministic, identical for every sample, and confined to the outer ⌊w/2⌋ positions; since $X_{v}=X-X_{u}$ holds exactly, whatever the padding assigns to the trend is compensated in the residual, so no information is lost, and the learned filters of both branches simply absorb this fixed edge pattern during training. Replicate and reflective padding were also tried and behave similarly in practice; zero padding was kept for its simplicity and because it keeps the decomposition a purely linear operator on the observed window.

#### 4.2.2. Spatial Feature Decomposition

Vehicle trajectories are constrained by the roads and travel directions encoded in the network, unlike movement in an unrestricted spatial domain. Let $down(v_{i})\subset V$ contain the intersections directly reachable from $v_{i}$. At time *t*, the proportion of flow leaving $v_{i}$ and continuing to $v_{j}\in down\left(v_{i}\right)$ is denoted by $W_{ij}^{G}$ and calculated using Equation (3).

(3) $$W_{ij}^{G}=\frac{X_{i\to j}^{t}}{X_{i}^{t}}.$$

In this expression, $X_{i}^{t}$ is the total outbound flow at $v_{i}$, and $X_{i\to j}^{t}$ is the portion transferred from $v_{i}$ to $v_{j}$. Collecting these proportions in $W_{G}$ describes how each intersection distributes traffic among its outgoing roads. When travel time along a road segment is ignored, the next-step flow at $v_{j}$ becomes the weighted sum of flows from its upstream neighbors, as stated in Equation (4).

(4) $$X_{j}^{t+1}=\sum_{v_{i}\in up\left(v_{j}\right)}W_{ij}^{G}X_{i}^{t}.$$

Because road graphs are sparse and directed operators are often less convenient, many prediction models replace *G* with the undirected first-order graph $G_{f}$. Its matrix $A_{G_{f}}$ is obtained by logically symmetrizing $A_{G}$: a pair of intersections is adjacent whenever at least one directed road exists between them. Equation (5) gives this construction.

(5) $$A_{G_{f}}\left(i,j\right)=A_{G}^{sym}\left(i,j\right)=\left\{\begin{array}{cc} 1, & \mathrm{if}\;A_{G}\left(i,j\right)=1\;\mathrm{or}\;A_{G}\left(j,i\right)=1,\\ 0, & \mathrm{otherwise}. \end{array}\right.$$

The condition $A_{G}\left(i,j\right)=1$ refers to the edge $\langle v_{i},v_{j}\rangle \in E$ introduced in Section 3.1. Symmetrization gives $A_{G_{f}}\left(i,j\right)=A_{G_{f}}\left(j,i\right)$, which simplifies computation but removes the distinction between upstream and downstream travel. Following Ref. [58], we recover part of this lost information without returning to a fully directed formulation. Two further undirected graphs are derived from $G_{f}$: the second-order in-degree graph $G_{i}$ and second-order out-degree graph $G_{o}$. Their matrices are specified in Equation (6).

(6) $$\left\{\begin{array}{c} A_{G_{i}}\left(i,j\right)=\sum_{k=1}^{N}\frac{A_{G_{f}}\left(k,i\right)\;A_{G_{f}}\left(k,j\right)}{\sum_{v=1}^{N}A_{G_{f}}\left(k,v\right)},\\ A_{G_{o}}\left(i,j\right)=\sum_{k=1}^{N}\frac{A_{G_{f}}\left(i,k\right)\;A_{G_{f}}\left(j,k\right)}{\sum_{v=1}^{N}A_{G_{f}}\left(v,k\right)}. \end{array}\right.$$

Equation (6) sums contributions from all possible intermediate intersections $v_{k}$. In $A_{G_{i}}$, the product $A_{G_{f}}\left(k,i\right)A_{G_{f}}\left(k,j\right)$ is non-zero when $v_{k}$ is connected as a common source to both $v_{i}$ and $v_{j}$. Dividing by $\sum_{v}A_{G_{f}}\left(k,v\right)$ reduces the contribution of high-degree sources, and summing over *k* gives the total shared-inflow association. The construction of $A_{G_{o}}$ exchanges source and destination roles by using $A_{G_{f}}\left(\cdot ,k\right)$ in place of $A_{G_{f}}\left(k,\cdot \right)$. Hence, $A_{G_{i}}\left(i,j\right)$ is large when $v_{i}$ and $v_{j}$ receive traffic from similar upstream locations, whereas $A_{G_{o}}\left(i,j\right)$ reflects common downstream destinations. The matrices $A_{G_{f}}$, $A_{G_{i}}$, and $A_{G_{o}}$ are positive semi-definite [58], so conventional spectral graph convolution can be applied to each. Together, $G_{i}$ and $G_{o}$ reintroduce source-sink information lost during symmetrization.

The degree normalization is important because a shared connection through a highly connected intersection should not carry the same specificity as a shared connection through a node with few links. Without normalization, hubs would dominate the second-order matrices simply because they participate in many paths. The weighted definitions instead emphasize shared sources and destinations that are more distinctive. Although $G_{i}$ and $G_{o}$ are undirected matrices, the way they are constructed remains sensitive to the direction of the original traffic relation and therefore preserves directional context in a form compatible with spectral convolution.

Because $G_{f}$, $G_{i}$, and $G_{o}$ are all derived from road topology, they cannot represent relationships absent from that topology. Yet unconnected intersections may behave similarly when they belong to the same commuting corridor or serve comparable urban functions. We therefore add the adaptive graph $G_{a}$, whose edges are estimated from data rather than prescribed in advance. Following common self-adaptive graph constructions, Equation (7) parameterizes $G_{a}$ with the trainable embedding matrix $E\in R^{N\times d}$. Its row $E_{i}$ is a *d*-dimensional latent representation of intersection $v_{i}$.

(7) $$A_{G_{a}}=ReLU\left(EE^{T}\right),\;A_{G_{a}}\left(i,j\right)=ReLU\;\left(\sum_{r=1}^{d}E_{ir}E_{jr}\right).$$

The inner product $E_{i}E_{j}^{T}=\sum_{r=1}^{d}E_{ir}E_{jr}$ in Equation (7) measures similarity between $v_{i}$ and $v_{j}$ in the learned latent space. Applying ReLU(·) removes negative affinities and retains non-negative edge weights. Since $E_{i}E_{j}^{T}=E_{j}E_{i}^{T}$, $A_{G_{a}}$ is symmetric as well as non-negative. Its degree matrix satisfies $D_{G_{a}}\left(i,i\right)=\sum_{j=1}^{N}A_{G_{a}}\left(i,j\right)$, and the normalized Laplacian $L_{G_{a}}=D_{G_{a}}^{-\frac{1}{2}}A_{G_{a}}D_{G_{a}}^{-\frac{1}{2}}$ is formed in the same manner as for the topology-based graphs. Consequently, the Chebyshev convolution in Equation (10) can process $G_{a}$ without alteration. In contrast to the fixed matrices $A_{G_{f}}$, $A_{G_{i}}$, and $A_{G_{o}}$, *E* is randomly initialized and updated through the forecasting objective. The adaptive graph therefore evolves toward latent relationships that support prediction and supplements the explicit connections in the other three views.

Joint optimization means that $G_{a}$ is not constructed by a separate similarity threshold or preprocessing rule. Gradients from forecast error update the embeddings directly, favoring edges that improve the final task rather than relationships that are merely correlated in the raw data. The graph may consequently represent shared daily demand, comparable functional roles, or other latent factors that are not observable from road geometry alone. At the same time, keeping $G_{a}$ as one view among four prevents learned affinity from replacing the known physical network.

The symmetry of $A_{G_{a}}=ReLU\left(EE^{T}\right)$ is a deliberate choice rather than a limitation, and it remains appropriate for directional traffic for several reasons. Within MPSTFFM, directional structure is already the responsibility of the topology-derived views: $G_{i}$ and $G_{o}$ separate shared-upstream from shared-downstream relations explicitly, so the adaptive view does not need to duplicate this role. The relationships $G_{a}$ is meant to capture—co-membership in a commuting corridor, comparable land use, synchronized daily demand—are also inherently mutual: if the behavior of $v_{i}$ is informative about $v_{j}$ through a shared latent factor, the converse holds by the same factor, so a symmetric affinity is the natural choice. Symmetry also provides guarantees the shared extraction machinery depends on: a symmetric, non-negative $A_{G_{a}}$ yields a normalized Laplacian with a real spectrum, so the Chebyshev convolution of Equation (10) applies to $G_{a}$ without modification, and all four graph views can be processed by the same operator, keeping their branch outputs directly comparable at fusion. An asymmetric alternative, such as two independent source and target embedding matrices $E_{1}E_{2}^{T}$, would need a directed propagation scheme for that one view, breaking this uniformity, doubling the embedding parameters, and reintroducing the same modeling burden the directional second-order graphs already carry. The single-embedding form also regularizes the learned graph: with N×d parameters instead of 2Nd, and d≪N, the rank-*d* structure of $EE^{T}$ limits $G_{a}$ to a small number of latent factors, which curbs overfitting on the sparse edge evidence available from forecast gradients. This division of labor matches what the ablations show: in the decomposition study of Section 6.3, configurations containing $G_{a}$ improve over their counterparts without it even when $G_{i}$ and $G_{o}$ are present, so the symmetric adaptive view is contributing information complementary to the directional views rather than duplicating them.

**Figure 3:**
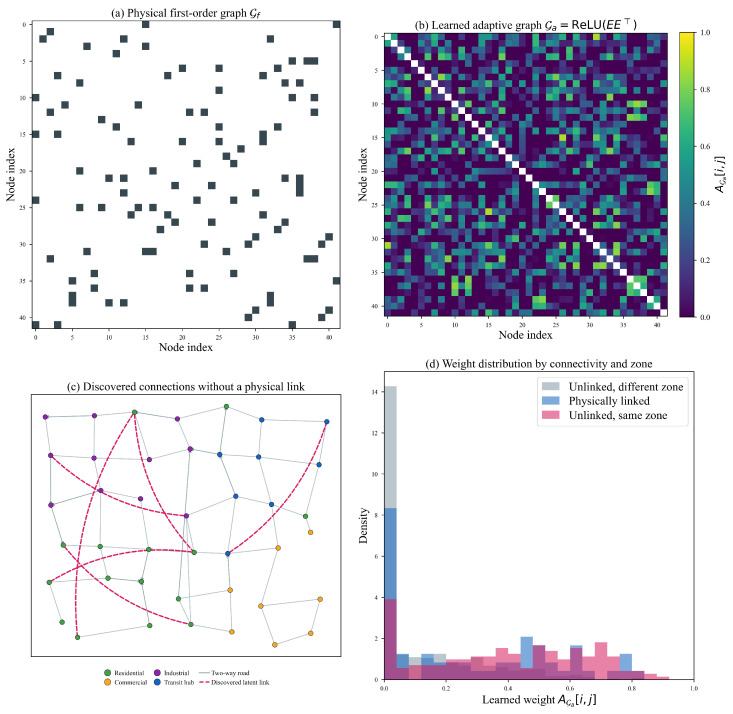
Illustration of what the adaptive graph mechanism captures, on a controlled synthetic sub-network of 42 nodes with a known latent structure (four functional zones: residential, commercial, industrial, transit hub). (**a**) Physical first-order graph $G_{f}$, mostly two-way with a minority of one-way streets. (**b**) Heatmap of the learned adjacency $A_{G_{a}}=ReLU\left(EE^{T}\right)$. (**c**) Spatial layout with physical roads in grey and the six node pairs that receive the largest $A_{G_{a}}$ weight among all pairs with no physical link, highlighted in pink. (**d**) Distribution of $A_{G_{a}}$ weights for physically linked pairs, unlinked pairs sharing a functional zone, and unlinked pairs from different zones.

Figure 3 shows what the learned adjacency $A_{G_{a}}$ actually captures. The trained embeddings *E* are tied to the anonymized sensor identifiers of the benchmark datasets used in Section 6, which carry no public functional labels such as land use or corridor membership against which a learned connection could be checked, so the mechanism of Equation (7) is instead illustrated on a controlled synthetic sub-network whose latent structure is known by construction: each node belongs to one of four functional zones, and node embeddings are generated so that nodes sharing a zone have high inner-product similarity regardless of spatial distance, reproducing the role *E* plays in $A_{G_{a}}=ReLU\left(EE^{T}\right)$. Figure 3a shows the physical graph $G_{f}$ for reference, and Figure 3b shows the corresponding heatmap of $A_{G_{a}}$, whose entries are dense and graded rather than sparse and binary, reflecting a continuum of learned similarity rather than a fixed topological relation. Figure 3c gives concrete examples of node pairs that turn out to be strongly connected despite having no direct road link: the six pairs with the largest $A_{G_{a}}$ weight among all physically unconnected, spatially distant pairs are drawn explicitly, and in every case the two endpoints share the same functional zone, so the adaptive graph is linking locations with similar latent function even where $G_{f}$, $G_{i}$, and $G_{o}$ provide no path between them. Figure 3d explains why the resulting improvement is modest rather than large: only a minority of unlinked same-zone pairs reach a high weight, most sit in an intermediate range that overlaps with the weights of physically linked pairs, and unlinked different-zone pairs are pushed toward zero. This matches the 2–3% MAE improvement attributed to $G_{a}$ in the decomposition study of Section 6.3—the adaptive graph recovers a specific, limited set of informative long-range relationships rather than acting as a dense shortcut across the whole network.

The spatial feature set is thus $\left\{G_{f},G_{i},G_{o},G_{a}\right\}$. Each member supplies a different propagation structure: three describe observable topological relations, and one captures latent dependence learned from traffic data. Their features are fused so that the final representation reflects several mechanisms of spatial evolution. Section 6 evaluates the contribution of the individual graphs.

The distinction among these graphs can also be viewed in terms of the evidence used to connect two intersections. $G_{f}$ uses the existence of a direct road, $G_{i}$ uses evidence of common upstream supply, and $G_{o}$ uses evidence of common downstream routing. $G_{a}$ does not require any of these topological conditions and instead relies on similarity learned from forecasting error. A pair of nodes may therefore be strongly related in one graph and weakly related in another. Maintaining separate branches preserves this distinction until the fusion stage.

### 4.3. Spatio-Temporal Feature Extraction

Traffic at a node is shaped by dependencies at two scales. Local correlations connect nearby time steps and adjacent or structurally related intersections. Global correlations link observations separated by longer periods or larger portions of the network. Preserving both is necessary because neither short-range propagation nor broad network context alone captures the complete traffic process.

For every (X,G), where $X\in \{X_{u},X_{v}\}$ and $G\in \{G_{f},G_{i},G_{o},G_{a}\}$, the STFF block combines attention and convolution to model these scales. Temporal and spatial self-attention operate across the full observation window and network to obtain global context. A GCN and temporal CNN then learn local dependencies from graph neighborhoods and consecutive observations.

The sequence of these operations is deliberate. Attention first reweights the input using information from the complete temporal or spatial extent, giving the subsequent local operators a context-aware representation. Graph and temporal convolution then impose locality and extract structured patterns from that representation. Reversing the roles would require attention to recover global relationships after local aggregation had already mixed neighboring information. The adopted order preserves the original long-range comparisons before applying neighborhood-based refinement.

#### 4.3.1. Extraction of Global Spatio-Temporal Correlation

Global attention is applied to $X\in R^{N\times C\times S}$ in two successive steps. Equation (8) first computes the temporal attention matrix $SAT_{t}$, which reweights the input to give $Y_{1}=XSAT_{t}$. Spatial attention is then evaluated from $Y_{1}$ through Equation (9). Multiplication by $SAT_{s}$ produces $Y_{2}=Y_{1}SAT_{s}$, whose features contain global information from both axes.

(8) $$\begin{array}{cc} SAT_{t} & =Softmax\left(ReLU\left(\left(XV_{1}U_{1}\right){\left(XV_{1}U_{1}\right)}^{T}+b_{1}\right)\right), \end{array}$$

(9) $$\begin{array}{cc} SAT_{s} & =Softmax\left(ReLU\left(\left(Y_{1}V_{2}U_{2}\right){\left(Y_{1}V_{2}U_{2}\right)}^{T}+b_{2}\right)\right). \end{array}$$

In Equations (8) and (9), $V_{1},V_{2}\in R^{C}$ perform channel-wise projection. The matrices $U_{1}\in R^{N\times N^{\prime }}$ and $U_{2}\in R^{S\times S^{\prime }}$ map node and sequence features into lower-dimensional spaces, while $b_{1}\in R^{S\times S}$ and $b_{2}\in R^{N\times N}$ are learned biases. ReLU(·) denotes rectified linear activation and ${\left(\cdot \right)}^{T}$ indicates transposition.

Through these attention weights, $Y_{1}$ and $Y_{2}$ retain informative signal components and reduce the influence of redundant or noisy observations. The next stage supplements this global context with short-range interactions between related intersections and neighboring time steps.

Temporal attention is useful when the most relevant observation is not the immediately preceding one, for example when traffic follows a repeated pattern within the input period. Spatial attention likewise permits a node to use information from remote parts of the network without waiting for repeated graph propagation. These matrices are learned for the current features, so the relative importance assigned to times and locations may vary across the trend and fluctuation branches and across the selected graph perspectives.

#### 4.3.2. Extraction of Local Spatio-Temporal Correlation

Local traffic dependence follows from spatial connectivity and persistence over adjacent time steps. Graph convolution aggregates information according to the selected road relation, whereas temporal convolution combines observations inside a short sliding interval.

The graph-convolution input is $Y_{1}$, which already contains the temporal context obtained in Section 4.3.1. For $G\in \{G_{f},G_{i},G_{o},G_{a}\}$, Equation (10) uses a Chebyshev polynomial of order *k* to gather information from the *k*-hop neighborhood without explicitly decomposing the graph Laplacian.

(10) $$G_{\theta }{*}_{G}Y_{1}=\sum_{k=0}^{K}\theta_{k}T_{k}\left(L_{G}\right)Y_{1}.$$

The symbol ${*}_{G}$ denotes convolution on G, and $G_{\theta }$ is the learnable filter represented by a truncated Chebyshev series. The basis is evaluated on $L_{G}=D_{G}^{-\frac{1}{2}}A_{G}D_{G}^{-\frac{1}{2}}$, where $A_{G}$ and $D_{G}$ are the adjacency and degree matrices. Its terms satisfy $T_{k}\left(x\right)=2xT_{k-1}\left(x\right)-T_{k-2}\left(x\right)$, with $T_{0}\left(x\right)=1$ and $T_{1}\left(x\right)=x$, and the coefficients $\theta_{k}$ are optimized during training.

Applying the activation gives $Y_{2}=ReLU\left(G_{\theta }{*}_{G}Y_{1}\right)$, so each node representation reflects its local structure in G. A Conv2d operation with kernel size 1×m subsequently acts along time and yields $P\in R^{S\times m}$, which combines features over *m* consecutive observations. The output $Y=Y_{2}P$ therefore contains the global dependencies learned by attention and the local relationships introduced by graph and temporal convolution.

The Chebyshev formulation controls spatial range through *K*: lower orders emphasize immediate relations, while higher orders include progressively more distant graph neighborhoods. Temporal range is controlled separately by *m*. This separation allows the model to adjust local spatial and temporal coverage without increasing network depth solely to reach a wider neighborhood. Because both operations follow attention, their outputs remain conditioned on global context even though the convolutions themselves are local.

#### 4.3.3. Extraction of Deep Spatio-Temporal Correlation

For a perspective (X,G), the complete sequence of global and local extraction operations is represented by the parametric function ψ(·) in Equation (11).

(11) Y=ψ(X,G,Θ),

where $\Theta =\{\theta_{k},U_{1},U_{2},V_{1},V_{2},b_{1},b_{2},P\}$ contains the trainable parameters of one extraction layer.

A traffic disturbance can propagate to remote nodes and later observations, whereas one application of ψ(·) covers only a bounded neighborhood. Stacking *L* layers enlarges the receptive field gradually and enables the network to represent higher-order non-linear interactions.

Successive layers also transform the meaning of the propagated features. The first layer operates close to the observed flow, while later layers combine already contextualized patterns and can represent interactions among several local and global effects. Depth must nevertheless remain moderate, since excessive stacking increases computation and may overfit the available data. The sensitivity analysis in Section 6 examines this trade-off empirically.

Let $Y^{\left(l\right)}$ be the output at depth *l*. Consecutive layers are linked by a residual connection, which provides a direct route for gradient propagation and reduces vanishing-gradient effects. The resulting update is given in Equation (12).

(12) $$Y^{\left(l\right)}=\psi \left(Y^{\left(l-1\right)},G,\Theta^{\left(l\right)}\right)+Y^{\left(l-1\right)}.$$

Starting from $Y^{\left(0\right)}=X$, repeated substitution of $Y^{\left(l-1\right)}=\psi \left(Y^{\left(l-2\right)},G,\Theta^{\left(l-1\right)}\right)$ into Equation (12) expands the recursion. If $\psi^{\left(l\right)}\left(X,G,\Theta^{\left(1\right)}\ldots \Theta^{\left(l\right)}\right)$ denotes *l* successive applications, the expansion yields Equation (13):

(13) $$\begin{array}{cc} Y^{\left(l\right)} & =\psi \left(\psi \left(Y^{\left(l-2\right)},G,\Theta^{\left(l-1\right)}\right),G,\Theta^{\left(l\right)}\right)+\psi \left(Y^{\left(l-2\right)},G,\Theta^{\left(l-1\right)}\right)\\ \; & =\psi \left(\left(\ldots \psi \left(\psi \left(X,G,\Theta^{\left(1\right)}\right),G,\Theta^{\left(2\right)}\right),\ldots \right),G,\Theta^{\left(l\right)}\right)+\ldots +\psi \left(X,G,\Theta^{\left(1\right)}\right)+X\\ \; & =\sum_{i=1}^{l}\psi^{\left(i\right)}\left(X,G,\Theta^{\left(1\right)}\ldots \Theta^{\left(i\right)}\right). \end{array}$$

Equation (13) shows that the final layer contains contributions associated with several effective depths rather than only the deepest transformation. The direct residual paths retain lower-order information, while the composed terms represent progressively wider and more non-linear dependencies. This mixture is useful for traffic data because immediate neighborhood effects and broader propagation patterns can both influence the same forecast. It also stabilizes optimization by allowing useful shallow features to reach the output even when deeper transformations are still being learned.

### 4.4. Multi-Perspective Spatio-Temporal Feature Fusion

The *l*-layer extractor is run independently for all eight pairs produced in Section 4.2. Crossing $\left\{X_{u},X_{v}\right\}$ with $\left\{G_{f},G_{i},G_{o},G_{a}\right\}$ gives $Y_{X_{u}G_{f}}^{\left(l\right)}$, $Y_{X_{u}G_{i}}^{\left(l\right)}$, $Y_{X_{u}G_{o}}^{\left(l\right)}$, $Y_{X_{u}G_{a}}^{\left(l\right)}$, $Y_{X_{v}G_{f}}^{\left(l\right)}$, $Y_{X_{v}G_{i}}^{\left(l\right)}$, $Y_{X_{v}G_{o}}^{\left(l\right)}$, and $Y_{X_{v}G_{a}}^{\left(l\right)}$. Each output encodes a different aspect of the same traffic state.

Fusion combines this complementary information into a more expressive representation. A learned scalar $\alpha_{i}$ adjusts the contribution of each perspective, after which the weighted features are concatenated along the channel dimension. Equation (14) defines the resulting embedding $Z^{\left(l\right)}$.

Concatenation is used instead of averaging because averaging would collapse the branch identity and could cancel features with different scales or meanings. The expanded channel dimension retains the output of every branch, while α provides a simple mechanism for reducing the influence of less useful views. The fully connected prediction layer can then learn interactions among the preserved channels rather than receiving a prematurely compressed summary.

(14) $$Z^{\left(l\right)}=ReLU\left(concat\left(\alpha ⊙(Y_{X_{u}G_{f}}^{\left(l\right)},Y_{X_{u}G_{i}}^{\left(l\right)},Y_{X_{u}G_{o}}^{\left(l\right)},Y_{X_{u}G_{a}}^{\left(l\right)},Y_{X_{v}G_{f}}^{\left(l\right)},Y_{X_{v}G_{i}}^{\left(l\right)},Y_{X_{v}G_{o}}^{\left(l\right)},Y_{X_{v}G_{a}}^{\left(l\right)})\right)\right).$$

In Equation (14), ⊙ means that the *i*-th element of α scales the corresponding perspective feature element-wise before concatenation.

## 5. Traffic Flow Prediction Based on MPSTFFM

### 5.1. Algorithm Implementation

The fused tensor $Z^{\left(l\right)}\in R^{N\times 8S}$ is supplied to a fully connected layer that produces $\hat{Y}\in R^{N\times H}$ for the next *H* observations. Equation (15) gives this output mapping, whose parameters are learned through the objective $f_{L}$.

(15) $$\hat{Y}=ReLU\left(FC\left(Z^{\left(l\right)}\right)\right).$$

The training loss is the mean absolute error (MAE) between predictions and observations:

(16) $$f_{L}=\frac{1}{NH}\sum_{i=1}^{N}\sum_{t=1}^{H}\left|X_{i}^{t}-{\hat{Y}}_{i}^{t}\right|.$$

In Equation (16), $X_{i}^{t}$ denotes the observed flow at $v_{i}$ for forecast step *t*, and ${\hat{Y}}_{i}^{t}$ is its predicted value.

Algorithm 1 describes the end-to-end forecasting process. Lines 2–5 prepare the trend and fluctuation components, construct the three topology-based graphs and $G_{a}$, and generate the Chebyshev basis used by graph convolution. Lines 6–9 apply Algorithm 2 to every one of the eight perspective pairs. In lines 10–14, the extracted features are weighted, concatenated, and mapped to $\hat{Y}$ by the output layer. The embeddings *E* defining $G_{a}$ are trained jointly with the remaining model parameters.

Algorithm 2 details the processing of one pair (X,G) across *L* layers, beginning with $Y^{\left(0\right)}=X$. Each layer first introduces long-range context through temporal and spatial attention. Chebyshev graph convolution and 1D temporal convolution then recover local dependencies, and a residual addition combines the new output with the layer input before the next iteration.

The same computational sequence is used for every perspective, but the branch inputs and learned parameters lead to different representations. For instance, a convolution on $G_{i}$ aggregates intersections connected through common upstream nodes, whereas the corresponding operation on $G_{a}$ follows learned affinity. Similarly, attention applied to $X_{u}$ operates on a smoother series than attention applied to $X_{v}$. Sharing the architectural pattern keeps the branch outputs compatible for fusion while still allowing specialization through their data and parameters.
**Algorithm 1:** MPSTFFM-P: Traffic Flow Prediction based on MPSTFFM**Require:** $X\in R^{N\times C\times S}$: traffic flow; *G*: traffic network; *w*: sliding window size; *K*: Chebyshev order; *L*: number of neural network layers.**Ensure:** $\hat{Y}\in R^{N\times H}$: predicted traffic flow for *H* time units in the future.▹Spatio-temporal feature representation  1: $\{X_{u},X_{v}\}\leftarrow$ temporal feature decomposition of *X* with window *w*
  2: $\{G_{f},G_{i},G_{o}\}\leftarrow$ spatial feature decomposition of *G*  3: $G_{a}\leftarrow$ adaptive graph $ReLU(EE^{T})$ from learnable node embeddings *E*  4: Ch[K]← Chebyshev polynomial basis of graph Laplacian▹Multi-perspective spatio-temporal feature learning  5: **for** $X\in \{X_{u},X_{v}\}$ and $G\in \{G_{f},G_{i},G_{o},G_{a}\}$
**do**  6:       $Y_{XG}^{\left(L\right)}\leftarrow STFF\left(X,G,Ch\left[K\right],L\right)$  7: **end for**▹Feature fusion and prediction  8: Initialize parameters α[8]  9: $Z^{\left(L\right)}\leftarrow ReLU\left(concat\left(\alpha ⊙\left(Y_{X_{u}G_{f}}^{\left(L\right)},Y_{X_{u}G_{i}}^{\left(L\right)},Y_{X_{u}G_{o}}^{\left(L\right)},Y_{X_{u}G_{a}}^{\left(L\right)},Y_{X_{v}G_{f}}^{\left(L\right)},Y_{X_{v}G_{i}}^{\left(L\right)},Y_{X_{v}G_{o}}^{\left(L\right)},Y_{X_{v}G_{a}}^{\left(L\right)}\right)\right)\right)$ 10: $\hat{Y}\leftarrow ReLU\left(FC\left(Z^{\left(L\right)}\right)\right)$ 11: Optimize parameters according to $f_{L}$ 12: **return**
$\hat{Y}$


**Algorithm 2:** STFF: Spatio-temporal feature fusion block**Require:** $X\in R^{N\times C\times S}$: temporal features of traffic flow; G: spatial features of traffic flow; Ch[K]: Chebyshev polynomial basis; *L*: number of layers.**Ensure:** $Y\in R^{N\times S}$: traffic flow with spatio-temporal features.  1: **function** STFF(X,G,Ch[K],L)  2:       Let $Y^{\left(0\right)}=X$  3:       **while**
l<L
**do**▹Global Spatial-Temporal Feature Fusion  4:             $SAT_{t}\leftarrow Softmax\left(ReLU\left(\left(Y^{\left(l-1\right)}U_{1}V_{1}\right){\left(Y^{\left(l-1\right)}U_{1}V_{1}\right)}^{T}+b_{1}\right)\right)$  5:             $Y_{1}\leftarrow Y^{\left(l-1\right)}\times SAT_{t}$  6:             $SAT_{s}\leftarrow Softmax\left(ReLU\left(\left(Y_{1}U_{2}V_{2}\right){\left(Y_{1}U_{2}V_{2}\right)}^{T}+b_{2}\right)\right)$  7:             $Y_{2}\leftarrow SAT_{s}\times Y_{1}$▹Local Spatial-Temporal Feature Fusion  8:             $Y_{3}\leftarrow ReLU\left(\sum_{k=0}^{K}\theta_{k}Ch\left[k\right]\left(L_{G}\right)Y_{2}\right)$  9:             $Y^{\left(l\right)}\leftarrow Conv2d\left(Y_{3},\left(1,m\right)\right)$▹Residual network connect 10:             $Y^{\left(l\right)}\leftarrow Y^{\left(l\right)}+Y^{\left(l-1\right)}$ 11:       **end while** 12:       **return**
$Y^{\left(l\right)}$ 13: **end function**

### 5.2. Algorithm Complexity Analysis

MPSTFFM incurs computation in three parts: input and graph preparation, correlation learning for every perspective, and final fusion with output prediction.

The topology-derived graphs depend only on the road network and may be constructed offline. By contrast, training updates the embedding matrix *E* of the adaptive graph. Its adjacency $A_{G_{a}}=ReLU\left(EE^{T}\right)$ must therefore be recalculated at every iteration in $O(N^{2}d)$ time. Once *K* is fixed, the Chebyshev bases for the three static graphs are also precomputable, whereas the basis associated with $G_{a}$ must be updated with its adjacency. Temporal decomposition remains an online preparation operation and costs O(NSw). The dominant stage learns spatio-temporal features for *R* perspectives and has complexity $O(RKS^{2}N^{2}CL)$ per iteration. Fusion and prediction add O(NSH). Because practical values of *w* and *H* are small compared with *N* and *S*, the overall cost is governed by $O(RKS^{2}N^{2}CL)$.

## 6. Experiment Analysis

### 6.1. Experimental Environment and Data

The implementation uses Python 3.12 and PyTorch 2.1.0. Training and inference are performed on a Linux cluster with four NVIDIA RTX 5090 GPUs. Public benchmarks are included in the evaluation. LargeST-SD and LargeST-GBA are the San Diego and Greater Bay Area subsets of LargeST [59]; both originate from the California Traffic Performance Measurement System and contain three years of observations. The other two datasets are the commonly used medium-scale PEMSD4 and PEMSD8 benchmarks [21]. Table 1 summarizes their statistics.

All four datasets go through the same preparation pipeline. Raw detector measurements are aggregated to a 5-min sampling interval, matching the native resolution of the PEMS benchmarks and the released form of LargeST. Missing observations, caused mainly by detector outages and transmission failures, are repaired before windowing: gaps shorter than one hour are filled by linear interpolation along the affected sensor’s time axis, while longer outages are replaced with the historical average of that sensor at the same time of day, computed over the training portion only. No sensors were removed, so the vertex counts in Table 1 correspond to the complete released networks. After imputation, a sliding window of stride one pairs each S=12-step input segment with its subsequent H=12-step target; these windows are built separately within each partition after the 6:2:2 split, so no input–target pair crosses a partition boundary and no test information leaks into training. The adjacency matrix $A_{G}$ is taken directly from the connectivity files distributed with each benchmark, and $G_{f}$, $G_{i}$, and $G_{o}$ are derived from it once, as described in Section 4.2.2. Z-score normalization is fitted on the training portion only, as noted with the rest of the training configuration below. Because flow values of zero occur during night hours and would make percentage errors undefined, MAPE is computed with the usual masking convention that excludes zero targets, while MAE and RMSE use all target values without masking. This same pipeline—imputation, windowing, and masking—was applied identically to every baseline model.

The data partitioning is chronological and precedes sliding-window construction. For each dataset, the continuous time axis is divided into three contiguous, temporally ordered blocks: the first 60% of timestamps constitute the training set, the subsequent 20% the validation set, and the final 20% the test set. Sliding windows of stride one are then generated independently within each block, and any window whose input or target segment would cross a block boundary is discarded. Under this protocol, no input–target pair spans two partitions, no observation is shared between windows assigned to different subsets, and no validation- or test-period value appears in any training window, in either the input or the target position. Although the three blocks are necessarily adjacent at their boundaries, as in any chronological division of a single continuous series, this adjacency involves no sample overlap and therefore introduces no temporal leakage. Randomness in the experimental pipeline is confined to mini-batch shuffling and parameter initialization, both governed by the reported seeds, and does not affect the assignment of time periods to subsets. Furthermore, the historical averages used to impute long detector outages are computed exclusively from the training block, so the imputation procedure cannot propagate information from later partitions into earlier ones. The same chronological protocol was applied uniformly to MPSTFFM and to all twelve baseline models.

Unless otherwise stated, the experiments use the hyperparameters in Table 2. Each dataset is chronologically partitioned into training, validation, and test sets in a 6:2:2 ratio, as detailed below, with the training portion processed in mini-batches of size BS.

The complete training configuration is reported here for reproducibility. All trainable parameters, including the node embeddings *E* of the adaptive graph, are optimized with Adam ($\beta_{1}=0.9$, $\beta_{2}=0.999$, $\epsilon =10^{-8}$) at an initial learning rate of $10^{-3}$, halved whenever validation MAE fails to improve for 10 consecutive epochs. Weight decay of $10^{-4}$ is applied for regularization, and gradients are clipped to a maximum $\ell_{2}$-norm of 5. Training runs for at most 200 epochs, with early stopping after 20 epochs without validation-MAE improvement, and the checkpoint with the lowest validation MAE is used for testing. Input data are standardized by z-score normalization, with mean and standard deviation computed only on the training portion and applied unchanged to validation and test; predictions are de-normalized before the error metrics are computed. Network weights use Xavier uniform initialization, biases are set to zero, and the adaptive-graph embeddings *E* are drawn from N(0,1). Reported results are averages over five runs with random seeds {1,2,3,4,5} controlling data shuffling and initialization; the standard deviation of test MAE across seeds stayed below 0.5% of the mean for every dataset, indicating stable convergence. The same optimization and normalization procedure was applied to every baseline under the unified protocol of Section 6.2.

In Table 2, BS is the mini-batch size, SK and TK are the spatial and temporal convolution kernel sizes, and *d* specifies the dimension of the node embeddings *E* used to construct $G_{a}$.

### 6.2. Baseline Models and Evaluation Metrics

The comparison includes twelve recent forecasting methods, organized into four methodological groups:

- Graph neural network-based methods: STFGNN [3], LSGCN [9], Auto-DSTSGN [14], STSGCN [16], STGODE [21], STG-NCDE [22], and AutoSTG [60].
- Attention mechanism-based methods: ST-Ware [33] and PDFormer [20].
- Methods combining graph neural networks and attention mechanisms: ASTGNN [23], and STJGCN [24].
- Spatio-temporal data pre-modeling processing methods: STD-MAE [57].

Consistent with the evaluation practice used by most baselines, performance is measured by mean absolute error (MAE), root mean square error (RMSE), and mean absolute percentage error (MAPE).

All baseline results in Table 3 and Table 4 were produced by us rather than taken from the original publications, because several baselines predate the LargeST benchmark and report no results on LargeST-SD or LargeST-GBA, and the remaining methods were originally evaluated under different splits and horizons. Each baseline was trained and tested with the official open-source implementation released by its authors; where none was available, the model was re-implemented from the paper’s description and checked by reproducing its published PEMSD4/PEMSD8 results to within the reported precision before being applied to the other datasets. Every method, including MPSTFFM, followed the same protocol: an input window of S=12 steps, a forecast horizon of H=12 steps, an identical 6:2:2 training/validation/test partition generated once per dataset and shared across all models, the same z-score normalization fitted only on the training portion, and identical MAE, RMSE, and MAPE computations on the de-normalized test predictions. Model-specific hyperparameters such as learning rate, hidden dimension, number of layers, and dropout were tuned for each baseline on the shared validation set, starting from the values recommended in its original publication, so no competitor was run under a configuration weaker than its published default. Early stopping on validation MAE and the same batch size and hardware environment (Section 6.1) were used throughout.

### 6.3. Effectiveness of Spatio-Temporal Feature Decomposition

We evaluate the contribution of the decomposed features through 60 PEMSD8 configurations. Four temporal settings are considered: $X_{u}$ alone, $X_{v}$ alone, the original undecomposed series, and the pair $\left\{X_{u},X_{v}\right\}$. The four graphs $G_{f}$, $G_{i}$, $G_{o}$, and $G_{a}$ produce $2^{4}-1=15$ non-empty spatial subsets. Combining the four temporal settings with these subsets yields 4×15=60 experiments. In the notation used for this study, “+” denotes an included feature, “-” denotes an excluded feature, and “o” identifies the original flow series.

**Figure 4:**
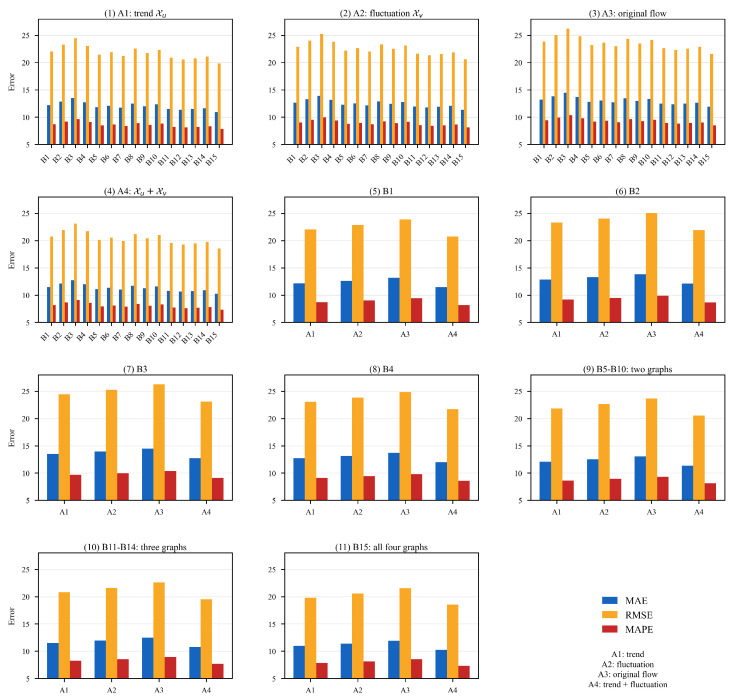
Results of the 60 spatio-temporal feature-decomposition configurations evaluated on PEMSD8.

Figure 4 presents the same experiments from two organizational perspectives. Strategy A groups configurations according to their temporal input, whereas strategy B groups them by spatial graph selection. Under strategy A, A4, which uses both decomposed components, gives the lowest errors; A3, based on the original series alone, gives the highest. This ordering supports the value of separating trend and fluctuation. Under strategy B, B15 performs best because it combines direct adjacency from $G_{f}$, shared inflow and outflow structures from $G_{i}$ and $G_{o}$, and latent associations from $G_{a}$. It also improves on B1, which contains only $G_{f}$. Among the single-graph settings, the $G_{o}$-only group is weakest because common destinations alone do not provide direct connectivity, shared-source information, or learned latent relations. The improvement obtained as complementary graphs are added suggests that the four spatial views contribute non-redundant information.

The two grouping strategies answer different questions. Strategy A compares temporal representations while repeatedly varying the available spatial evidence, making its ranking less dependent on one particular graph. Strategy B performs the converse comparison and shows how a spatial subset behaves under several temporal inputs. Agreement between the two views strengthens the interpretation: the advantage of A4 is not confined to one graph combination, and the benefit of B15 is not produced by only one temporal setting. The 60-run design therefore evaluates decomposition more systematically than an ablation that removes one feature from a single default configuration.

### 6.4. Comparison and Result Analysis

All models, including every baseline, are evaluated under the identical protocol described in Section 6.2, so the reported differences come from the architectures themselves rather than from data preparation, splitting, or metric computation. The input and output lengths are both 12 time steps, corresponding to one hour, and the data split is 6:2:2. Table 3 and Table 4 report the results, with the minimum error in each column shown in bold. MPSTFFM is ranked first for every metric on every dataset. The aggregate improvement is computed as the relative gain $\left(e_{\mathrm{best}}-e_{\mathrm{MPSTFFM}}\right)/e_{\mathrm{best}}$ for each dataset-metric pair, where $e_{\mathrm{best}}$ is the lowest error achieved by any of the twelve baselines on that dataset and metric in Table 3 and Table 4, and $e_{\mathrm{MPSTFFM}}$ is the corresponding error of the proposed model. Averaged over the four datasets, this gives mean reductions of 13.04% in MAE, 5.28% in RMSE, and 9.59% in MAPE relative to the strongest baseline.

Using a common protocol is necessary for a meaningful comparison because sequence length and data partition can materially affect reported error. The 12-step history gives each method the same amount of recent information, and the 12-step target evaluates a full one-hour forecast rather than a single near-term point. Applying the same split ratio also prevents differences in training-set size from being mistaken for architectural improvement. The tables therefore compare models under aligned input, output, and sampling conditions.

The seven methods based only on GNNs achieve broadly similar results and generally rank below the other categories. Within the attention group, PDFormer benefits from representing both local geographic distance and global semantic similarity, and consequently performs better than the geometry-independent ST-Ware. Hybrid models such as STJGCN and ASTGNN combine neighborhood aggregation with global attention and are stronger overall than most purely graph-based or purely attention-based alternatives. STD-MAE adopts a different strategy. It trains a masked-reconstruction autoencoder to improve the input and then feeds the reconstructed data to the existing GWNet architecture [61]. Its competitive performance shows that improving data representation can be as valuable as modifying the forecasting network itself.

These rankings also clarify why combining GNNs and attention is useful. Graph propagation is effective for fine-grained local structure, but even deeper networks or larger convolution supports reach distant nodes only through repeated neighborhood aggregation. Attention directly compares locations across the network and therefore captures long-range relationships, although it provides a weaker structural prior for immediate adjacency. MPSTFFM assigns these roles to different components: self-attention learns global context, graph convolution encodes local spatial relations, and temporal convolution represents local sequential behavior. The multi-perspective decomposition further exposes patterns that a single input view may hide. Their combined effect accounts for the model’s consistent advantage over the twelve baselines.

The consistency across datasets is also relevant because the benchmarks differ markedly in scale. LargeST-GBA contains far more vertices than PEMSD8, while the PEMS datasets cover shorter observation periods. A method that relies on one narrow form of dependency may not retain its ranking when network size or sampling context changes. MPSTFFM remains competitive across these conditions because its local operators exploit explicit graph structure and its attention modules provide a route to wider context without requiring the same number of propagation steps for every network.

### 6.5. Ablation Study

To isolate the effect of the principal MPSTFFM components, we compare the complete model with six ablated variants on LargeST-SD, PEMSD4, and PEMSD8:

- MPSTFFM w/o GT: removes the temporal self-attention mechanism, isolating the effect of global temporal dependencies.
- MPSTFFM w/o GS: removes the spatial self-attention mechanism, isolating the effect of global spatial dependencies.
- MPSTFFM w/o LS: removes the graph convolutional network, isolating the effect of local spatial aggregation.
- MPSTFFM w/o LT: removes the temporal convolutional network, isolating the effect of local temporal aggregation.
- MPSTFFM w/o AG: removes the adaptive spatial graph $G_{a}$, retaining only the three topology-derived graphs $\left\{G_{f},G_{i},G_{o}\right\}$, to isolate the contribution of the learned latent spatial dependencies.
- MPSTFFM w/o MPST: removes multi-perspective decomposition, replacing it with the single perspective formed by the original traffic flow and the base topology graph, to examine the effect of multi-perspective feature fusion.

**Figure 5:**
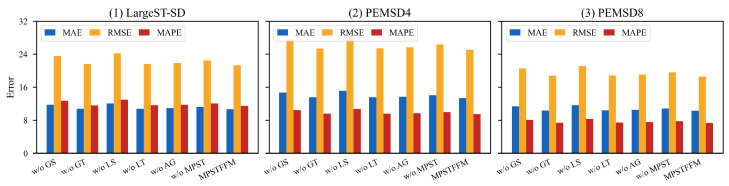
Results of ablation experiments conducted on the datasets LargeST-SD, PEMSD4, and PEMSD8.

Figure 5 compares the six variants with the complete model. Five observations follow from the results.

Relative importance of feature extraction components:The component ranking is stable across the three datasets. The largest deterioration occurs after removing spatial self-attention (w/o GS) or graph convolution (w/o LS). Excluding the complete multi-perspective decomposition (w/o MPST) produces an intermediate loss and performs slightly worse than removing only the adaptive graph (w/o AG). By comparison, the w/o GT and w/o LT variants remain closest to the full model. Spatial extraction is therefore the dominant contributor, while multi-perspective information provides an additional gain and temporal extraction has a smaller supporting effect.

Local versus global spatial feature extraction: The w/o LS variant is consistently less accurate than w/o GS. Thus, removing graph-based neighborhood aggregation is more harmful than removing global spatial attention. For these datasets, immediate structural relations contain more predictive information than network-wide spatial context.

Local versus global temporal feature extraction: LargeST-SD and PEMSD4 show almost no difference between w/o GT and w/o LT. On PEMSD8, w/o GT is only slightly better than w/o LT. Local temporal convolution may therefore have a small advantage over temporal attention, but the narrow gaps indicate that the two components make broadly similar contributions.

Contribution of multi-perspective decomposition: Replacing the decomposed inputs with one original-flow and base-graph perspective reduces accuracy on every dataset. The trend, fluctuation, and four spatial relations therefore provide complementary features that the single-view alternative does not reproduce.

Contribution of the adaptive spatial graph: Removing $G_{a}$ increases MAE by approximately 2–3% on each dataset. The w/o AG model remains stronger than w/o MPST but weaker than the complete architecture. Thus, learned links between behaviorally related and topologically distant intersections add useful information, although their marginal value is smaller than that of the fixed graphs. This relatively modest gain is plausible for sparse, mainly unidirectional networks in which direct neighborhoods already explain much of the spatial variation.

The dataset structure helps explain the ablation ordering. Within the one-hour input period, flow at a single node changes only moderately from one step to the next, whereas upstream-to-downstream routing creates persistent spatial relationships. Because the graphs are sparse and largely unidirectional, a node’s traffic often resembles that of its immediate topological neighbors, increasing the importance of local graph convolution. Sparsity also reduces the number of informative second-order in-degree and out-degree links and limits the gain from directional decomposition. The learned edges of $G_{a}$ are not bound by this constraint and may connect distant locations with similar behavior, as reflected by the w/o AG comparison. Temporal decomposition adds less information because short-term variation within the input hour is comparatively limited. Accordingly, multi-perspective fusion improves all three datasets, but the quality of spatial modeling has the greater influence.

This interpretation does not imply that temporal processing is unnecessary. The small degradation of w/o GT and w/o LT is measured relative to a model that still receives a one-hour sequence and retains the remaining temporal operation. Each ablation removes only one mechanism, so the result describes marginal contribution rather than the value of the complete temporal dimension. Similarly, the gain from $G_{a}$ is evaluated in the presence of three strong topology-derived graphs. Its modest incremental effect should therefore be understood as complementary to, rather than independent of, the fixed spatial structure.

Why temporal decomposition helps. The raw flow series superimposes two processes with different statistical character: a smooth, strongly autocorrelated component driven by recurring demand, and a low-magnitude, weakly autocorrelated residual driven by disturbances. A single set of temporal filters has to trade off between them—filters smooth enough to track the trend attenuate the residual, while filters responsive enough to follow the residual inject its variance into the trend estimate. Decomposition removes this trade-off: the trend branch works on a signal whose step-to-step changes are small, so its attention and convolution parameters can specialize in slowly varying structure, while the fluctuation branch learns the dynamics of deviations around that stable reference. Since $X_{u}+X_{v}=X$ exactly, no information is discarded—the model simply receives the same content in a form better suited to specialized filters. Figure 4 shows this directly: configuration A4 (both components) outperforms A3 (the raw series) and both single-component settings (A1, A2) across all fifteen spatial subsets, so the gain comes from the separation itself rather than from any particular graph combination.

Why multiple graph representations help. Each graph defines a different propagation operator, and graph convolution can only mix information along the edges that operator provides. On $G_{f}$ alone, two intersections that share an upstream source but have no direct link only exchange information after two propagation steps through the intermediate node, diluted by everything else that node aggregates. The second-order graphs $G_{i}$ and $G_{o}$ turn these shared-source and shared-destination relations into direct edges, so correlated inflow and outflow patterns are aggregated in a single convolution step, with weights that preserve the directional origin that symmetrizing $G_{f}$ had erased. The four operators are therefore not redundant re-weightings of the same neighborhood but access paths to different second-order structure—consistent with the steady improvement in Figure 4 as graphs are added (B1 through B15) and the intermediate loss of the w/o MPST variant in Figure 5.

Why adaptive graph learning helps. The three topology-derived graphs are limited to relations that physical connectivity can express, yet traffic at topologically distant intersections can still be correlated through shared demand generators—a commuting corridor, an event venue, comparable land use—that leave no trace in the road graph. Because $G_{a}$ is optimized by forecast-error gradients rather than a preprocessing similarity rule, an edge survives in it only if propagating information along it actually reduces prediction error, which biases the learned graph toward relations that are predictive rather than merely correlated. This matches the consistent but moderate 2–3% MAE degradation seen for w/o AG: on freeway networks, where immediate topology already explains most of the spatial variance, latent long-range relations act mainly as a corrective supplement, and their contribution should grow in denser urban grids where functional similarity diverges more from physical adjacency.

Together, these three elements address complementary failure modes of a single-view model—temporal filter interference, restricted propagation paths, and topology-bounded relations—and the fusion weights α let the model arbitrate their contributions for each dataset. This is why the full model exceeds every ablated variant even though no single component is indispensable on its own.

### 6.6. Parameters Sensitivity Study

Parameter sensitivity is examined on PEMSD8 to guide the practical configuration of MPSTFFM. One parameter is varied at a time: the decomposition window *w*, Chebyshev order *K*, network depth *L*, spatial kernel size SK, temporal kernel size TK, and prediction horizon *H*.

**Figure 6:**
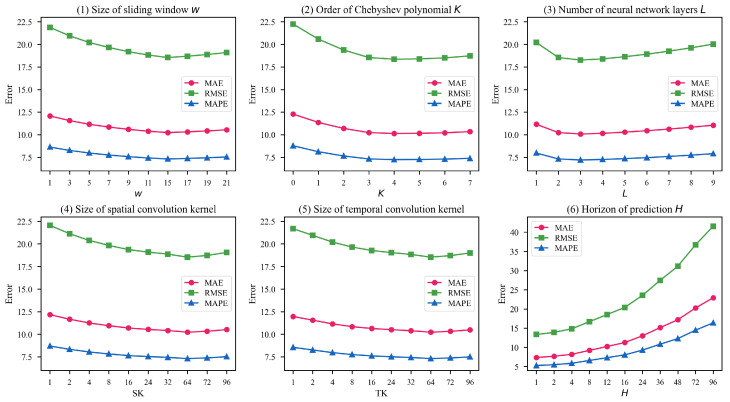
Results of parameter sensitivity experiments on the dataset PEMSD8.

Figure 6(1) evaluates the averaging window *w*. Performance improves as the window grows and is best at w=15, after which it decreases gradually. A 15-step interval removes transient noise without eliminating meaningful short-term changes. Smaller windows leave excessive variation in the trend, whereas larger windows smooth genuine demand movements into the residual. Both cases weaken the temporal decomposition.

The influence of *K* is shown in Figure 6(2). Increasing the polynomial order initially improves the approximation of the graph spectral filter and strengthens local spatial extraction, consistent with the importance of local structure observed in the ablation study. Improvement becomes small near K=4 and may reverse at larger orders. High-order terms describe increasingly fine spectral variation, which may contain less stable traffic information and amplify measurement noise.

Figure 6(3) varies the depth *L*. One layer provides a narrow receptive field and limited non-linear capacity. Additional layers broaden the accessible spatio-temporal context, with marked gains up to L=3. Beyond this point, the additional capacity yields little benefit and increases overfitting risk, causing validation performance to level off or deteriorate. Depths of two or three layers therefore offer the most effective balance.

Figure 6(4),(5) report the results for SK and TK. In both dimensions, enlarging the kernel from 1 initially improves accuracy because a broader local region contributes to each feature. The best results occur near 64. Larger kernels add parameters and begin to combine locations or periods with weaker semantic relevance, so their additional coverage produces diminishing returns and eventually a gradual loss of accuracy.

The forecast horizon analysis in Figure 6(6) holds the input length at 12 steps (12×5 min = 1 h) and changes *H* from 1 to 96. Accuracy remains high while the target lies within approximately the same temporal span as the observed history. Beyond 12 steps, current observations provide progressively less information about the target and forecast uncertainty accumulates. Errors consequently increase with *H*, particularly when the prediction interval becomes much longer than the input window.

Taken together, the sensitivity results support the default values in Table 2. The selected configuration lies near the best region for each parameter without relying on unnecessarily high polynomial order, depth, or kernel size. This balance is important because a small accuracy gain from a larger setting may not justify its additional parameters and computation. The experiments therefore identify not only the location of the minima but also the ranges over which performance remains comparatively stable.

## 7. Conclusions

This paper introduced MPSTFFM for urban traffic forecasting through multi-perspective feature learning. The temporal input is divided into trend and fluctuation components, and the road system is represented by adjacency, in-degree, out-degree, and adaptive graphs. These views jointly describe physical connectivity, directional flow relationships, and latent associations not contained in the fixed topology. Within every temporal–spatial pair, self-attention learns long-range dependence, while GCN and temporal CNN operations extract local structure. The eight outputs are fused into a common high-dimensional representation. This design preserves known road constraints and supplements them with data-driven links between locations whose behavior is correlated. Evaluation on public datasets showed that MPSTFFM outperformed twelve recent baselines, supporting the use of complementary spatial and temporal representations. Future research may incorporate weather, incidents, calendar information, and other external context into the same framework to improve adaptation across a broader range of operating conditions.

The present framework also has limitations worth acknowledging. Processing eight perspectives through parallel branches with unshared parameters multiplies the computational and memory footprint relative to single-view models, and the global temporal attention scales quadratically with the input length, so the framework is heavier to train than compact baselines such as Graph WaveNet; its advantage has to be weighed against this cost in resource-constrained deployments. Key structural hyperparameters—the moving-average width *w*, the input and forecast horizons, and the embedding dimension *d* of the adaptive graph—are also fixed globally rather than adapted per sensor or per period, even though traffic regimes differ between arterial and peripheral locations and between weekdays and weekends. The adaptive graph is further constrained to a symmetric, rank-*d* affinity; Section 4.2.2 argues this is an appropriate division of labor given the directional second-order views, but it also means that latent asymmetric long-range relations, if they exist, cannot be represented by any view. The model relies on flow history alone, so exogenous factors known to shape urban traffic—weather, incidents, public events, signal timing—are not incorporated, which limits accuracy in exactly the disturbance-driven situations where prediction matters most. Finally, the evaluation is confined to Californian freeway benchmarks, and whether the observed advantages transfer to dense signalized urban grids, where physical topology and functional correlation relate quite differently, remains to be shown. These limitations motivate the future directions outlined below.

The experimental analysis further indicates that spatial representation is especially important for the studied networks. Local graph aggregation produces the largest marginal contribution, while the adaptive graph and temporal decomposition provide smaller but consistent improvements. These findings support a balanced design in which fixed topology remains the main structural prior and learned relationships extend rather than replace it. They also suggest that the value of each perspective may change for denser networks, longer input histories, or datasets with stronger external disturbances, which provides a basis for broader future evaluation.

Accordingly, the proposed framework should be understood as a flexible and broadly applicable representation scheme rather than a fixed claim that every perspective contributes equally under all considered network conditions.

## Figures and Tables

**Table 1:** Description of the experimental dataset.

Datasets	Vertices	Edges	Timestamps	Time Range
LargeST-SD	716	1732	315,648	1 January 2019–31 December 2021
PEMSD4	307	340	16,992	1 January 2018–28 February 2018
LargeST-GBA	2352	5020	315,648	1 January 2019–31 December 2021
PEMSD8	170	295	17,856	1 July 2016–31 August 2016

**Table 2:** Default values for model parameters and training settings.

Parameters	*w*	*S*	*H*	*K*	*L*	BS	SK	TK	*d*
Value	15	12	12	3	2	32	64	64	10
Parameters	Optimizer	LR	Decay	Clip	Epochs	Patience	Seeds	Init	Norm
Value	Adam	$10^{-3}$	$10^{-4}$	5	200	20	5	Xavier	z-score

**Table 3:** Comparison of experimental results of models on datasets LargeST-SD and PEMSD4.

	LargeST-SD			PEMSD4		
Model	RMSE	MAE	MAPE(%)	RMSE	MAE	MAPE(%)
STSGCN	28.32	18.25	17.55	32.41	22.11	14.67
LSGCN	28.90	18.82	17.83	32.75	22.71	14.22
AutoSTG	26.92	17.45	15.32	31.66	21.82	13.50
STFGNN	27.91	15.88	17.82	31.25	21.72	15.44
STGODE	26.30	15.71	16.82	30.98	21.20	12.97
Auto-DSTSGN	26.50	13.25	14.36	31.60	17.91	13.43
STG-NCDE	26.10	14.60	14.24	32.11	18.32	11.79
ST-aware	26.25	16.40	13.72	30.34	18.12	12.40
ASTGNN	25.50	14.27	16.70	29.05	17.41	11.25
PDFormer	24.35	13.21	14.20	28.26	14.24	10.23
STJGCN	24.51	13.33	13.75	29.20	17.91	10.71
STD-MAE	23.77	12.93	12.88	26.44	15.36	10.80
MPSTFFM	21.32	10.62	11.45	25.05	13.36	9.47

**Table 4:** Comparison of experimental results of models on datasets LargeST-GBA and PEMSD8.

	LargeST-GBA			PEMSD8		
Model	RMSE	MAE	MAPE(%)	RMSE	MAE	MAPE(%)
STSGCN	37.41	25.32	11.33	25.71	18.21	11.82
LSGCN	39.92	26.55	12.51	25.22	18.40	12.42
AutoSTG	35.80	22.30	10.81	24.50	15.22	11.55
STFGNN	35.21	24.33	9.87	25.40	15.82	10.24
STGODE	36.45	21.72	11.26	24.80	15.72	10.23
Auto-DSTSGN	32.21	19.18	9.72	22.23	13.30	9.78
STG-NCDE	32.72	19.72	9.71	23.72	14.45	9.83
ST-aware	32.24	19.25	8.25	23.27	14.27	8.88
ASTGNN	25.78	18.92	8.73	22.92	13.29	9.71
PDFormer	25.92	17.90	8.91	21.72	12.90	9.91
STJGCN	28.72	18.83	8.42	22.36	13.92	9.24
STD-MAE	27.91	17.95	7.92	20.21	12.91	8.80
MPSTFFM	26.48	16.57	7.69	18.54	10.23	7.31

## Data Availability

All datasets used in the experiments are publicly available. The LargeST benchmark, including its SD and GBA subsets, can be found at https://github.com/liuxu77/LargeST (10 May 2025), while PEMSD4 and PEMSD8 are available through the STGODE repository at https://github.com/square-coder/STGODE (27 June 2025) and the standard PEMS benchmark releases. All data originate from the California Department of Transportation’s Performance Measurement System (PeMS, https://pems.dot.ca.gov (21 September 2025)).

## References

[1] Tedjopurnomo D.A., Bao Z., Zheng B., Choudhury F.M., Qin A.K. (2022). A survey on modern deep neural networks for traffic prediction: Trends, methods and challenges. IEEE Trans. Knowl. Data Eng..

[2] Jin G., Liang Y., Fang Y., Shao Z., Huang J., Zhang J., Zheng Y. (2023). Spatio-temporal graph neural networks for predictive learning in urban computing: A survey. IEEE Trans. Knowl. Data Eng..

[3] Li M., Zhu Z. (2021). Spatial-temporal fusion graph neural networks for traffic flow forecasting. Proc. AAAI Conf. Artif. Intell..

[4] Liang Y., Ouyang K., Jing L., Ruan S., Liu Y., Zhang J., Rosenblum D.S., Zheng Y. (2019). UrbanFM: Inferring fine-grained urban flows. Proceedings of the 25th ACM SIGKDD International Conference on Knowledge Discovery and Data Mining, Anchorage, AK, USA, 4–8 August 2019.

[5] Zhang J., Zheng Y., Qi D. (2017). Deep spatio-temporal residual networks for citywide crowd flows prediction. Proc. AAAI Conf. Artif. Intell..

[6] Wu Y., Tan H., Qin L., Ran B., Jiang Z. (2018). A hybrid deep-learning-based traffic flow prediction method and its understanding. Transp. Res. Part C Emerg. Technol..

[7] Li Y., Yu R., Shahabi C., Liu Y. (2018). Diffusion convolutional recurrent neural network: Data-driven traffic forecasting. Proceedings of the International Conference on Learning Representations.

[8] Zhao L., Song Y., Zhang C., Liu Y., Wang P., Lin T., Deng M., Li H. (2020). T-GCN: A temporal graph convolutional network for traffic prediction. IEEE Trans. Intell. Transp. Syst..

[9] Huang R., Huang C., Liu Y., Dai G., Kong W. (2021). LSGCN: Long short-term traffic prediction with graph convolutional networks. Proceedings of the Twenty-Ninth International Joint Conference on Artificial Intelligence.

[10] Zhong R., Hu B., Wang F., Feng Y., Li Z., Song X., Wang Y., Lou S., Tan J. (2025). Multi-factor embedding GNN-based traffic flow prediction considering intersection similarity. Neurocomputing.

[11] Huang Y., Ying J.J.-C., Tseng V.S. (2021). Spatio-attention embedded recurrent neural network for air quality prediction. Knowl. Based Syst..

[12] Lu H., Ge Z., Song Y., Jiang D., Zhou T., Qin J. (2021). A temporal-aware LSTM enhanced by a loss-switch mechanism for traffic flow forecasting. Neurocomputing.

[13] Ma C., Dai G., Zhou J. (2022). Short-term traffic flow prediction for urban road sections based on time-series analysis and the LSTM-BiLSTM method. IEEE Trans. Intell. Transp. Syst..

[14] Jin G., Li F., Zhang J., Wang M., Huang J. (2023). Automated dilated spatio-temporal synchronous graph modeling for traffic prediction. IEEE Trans. Intell. Transp. Syst..

[15] Zhao W., Zhang S., Zhou B., Wang B. (2023). Multi-spatio-temporal fusion graph recurrent network for traffic forecasting. Eng. Appl. Artif. Intell..

[16] Song C., Lin Y., Guo S., Wan H. (2020). Spatial-temporal synchronous graph convolutional networks: A new framework for spatial-temporal network data forecasting. Proc. AAAI Conf. Artif. Intell..

[17] Huang X., Ye Y., Yang X., Xiong L. (2023). Multi-view dynamic graph convolution neural network for traffic flow prediction. Expert Syst. Appl..

[18] Jin D., Shi J., Wang R., Li Y., Huang Y., Yang Y.-B. (2023). Trafformer: Unify time and space in traffic prediction. Proc. AAAI Conf. Artif. Intell..

[19] Xiao G., Tong H., Shu Y., Ni A. (2025). Spatial-temporal load prediction of electric bus charging stations based on S2TAT. Int. J. Electr. Power Energy Syst..

[20] Jiang J., Han C., Zhao W.X., Wang J. (2023). PDFormer: Propagation delay-aware dynamic long-range transformer for traffic flow prediction. Proc. AAAI Conf. Artif. Intell..

[21] Fang Z., Long Q., Song G., Xie K. (2021). Spatial-temporal graph ODE networks for traffic flow forecasting. Proceedings of the 27th ACM SIGKDD Conference on Knowledge Discovery and Data Mining, Singapore, 14–18 August 2021.

[22] Choi J., Choi H., Hwang J., Park N. (2022). Graph neural controlled differential equations for traffic forecasting. Proc. AAAI Conf. Artif. Intell..

[23] Guo S., Lin Y., Wan H., Li X., Cong G. (2022). Learning dynamics and heterogeneity of spatial-temporal graph data for traffic forecasting. IEEE Trans. Knowl. Data Eng..

[24] Zheng C., Fan X., Pan S., Jin H., Peng Z., Wu Z., Wang C., Yu P.S. (2024). Spatio-temporal joint graph convolutional networks for traffic forecasting. IEEE Trans. Knowl. Data Eng..

[25] Chan K.Y., Dillon T.S., Singh J., Chang E. (2012). Neural-network-based models for short-term traffic flow forecasting using a hybrid exponential smoothing and Levenberg–Marquardt algorithm. IEEE Trans. Intell. Transp. Syst..

[26] Guo J., Huang W., Williams B.M. (2014). Adaptive Kalman filter approach for stochastic short-term traffic flow rate prediction and uncertainty quantification. Transp. Res. Part C Emerg. Technol..

[27] Hou Y., Edara P., Chang Y. (2017). Road network state estimation using random forest ensemble learning. Proceedings of the IEEE International Conference on Intelligent Transportation Systems.

[28] Habtemichael F.G., Cetin M. (2016). Short-term traffic flow rate forecasting based on identifying similar traffic patterns. Transp. Res. Part C Emerg. Technol..

[29] Wu Y., Tan H. (2016). Short-term traffic flow forecasting with spatial-temporal correlation in a hybrid deep learning framework. arXiv.

[30] Kong W., Guo Z., Liu Y. (2024). Spatio-temporal pivotal graph neural networks for traffic flow forecasting. Proc. AAAI Conf. Artif. Intell..

[31] Wang T., Ngoduy D., Zou G., Dantsuji T., Liu Z., Li Y. (2024). PI-STGnet: Physics-integrated spatiotemporal graph neural network with a fundamental diagram learner for highway traffic flow prediction. Expert Syst. Appl..

[32] Hu J., Zheng T., Peng L., Teng F., Du S., Li T. (2025). LightST: A simplifying spatio-temporal graph neural network for traffic flow forecasting. IEEE Trans. Big Data.

[33] Cirstea R.-G., Yang B., Guo C., Kieu T. Towards spatio-temporal-aware traffic time-series forecasting. Proceedings of the IEEE 38th International Conference on Data Engineering.

[34] Chen J., Zheng L., Hu Y., Wang W., Zhang H., Hu X. (2024). Traffic flow matrix-based graph neural network with an attention mechanism for traffic flow prediction. Inf. Fusion.

[35] Yu Q., Ding W., Zhang H., Yang Y., Zhang T. (2024). Rethinking attention mechanisms for spatio-temporal modeling: A decoupling perspective in traffic flow prediction. Proceedings of the 33rd ACM International Conference on Information and Knowledge Management, Boise, ID, USA, 21–25 October 2024.

[36] Alsehaimi B., Alzamzami O., Alowidi N., Ali M. (2025). An adaptive spatio-temporal traffic flow prediction using self-attention and multi-graph networks. Sensors.

[37] Jeon S.-B., Jeong M.-H. (2024). Integrating spatio-temporal graph convolutional networks with convolutional neural networks for predicting short-term traffic speed in urban road networks. Appl. Sci..

[38] Singh V., Sahana S.K., Bhattacharjee V. (2024). Integrated spatio-temporal graph neural network for traffic forecasting. Appl. Sci..

[39] Karthika B., Uma Maheswari N. (2025). Enhanced multi-objective graph learning approach for optimizing traffic speed prediction on spatial and temporal features. Sci. Rep..

[40] Anitha G., Loganathan K., Srinivas Rao K. (2025). An efficient intelligent transportation system for traffic flow prediction using meta-temporal hyperbolic quantum graph neural networks. Sci. Rep..

[41] Mundada T., Ramdhave S., Jain S., Gupta S. (2026). Federated spatial-temporal traffic forecasting with VMD-enhanced graph attention and LSTM. Sci. Rep..

[42] Li Z., Xia L., Tang J., Xu Y., Shi L., Xia L., Yin D., Huang C. (2024). UrbanGPT: Spatio-Temporal Large Language Models.

[43] Liu C., Yang S., Xu Q., Li Z., Long C., Li Z., Zhao R. Spatial-temporal large language model for traffic prediction. Proceedings of the 25th IEEE International Conference on Mobile Data Management.

[44] Xu Y., Liu M. (2025). GPT4TFP: Spatio-temporal fusion large language model for traffic flow prediction. Neurocomputing.

[45] Guo X., Zhang Q., Jiang J., Peng M., Zhu M., Yang H.F. (2024). Towards explainable traffic flow prediction with large language models. Commun. Transp. Res..

[46] Rong Y., Mao Y., Cui H., He X., Chen M. (2024). Edge-computing-enabled large-scale traffic flow prediction with GPT in an intelligent autonomous transport system for a 6G network. IEEE Trans. Intell. Transp. Syst..

[47] Jiang F., Han X., Wen S., Tian T. (2025). Spatiotemporal interactive learning dynamic adaptive graph convolutional network for traffic forecasting. Knowl.-Based Syst..

[48] Zhang D., Wang P., Ding L., Wang X., He J. (2025). Spatio-temporal contrastive-learning-based adaptive graph augmentation for traffic flow prediction. IEEE Trans. Intell. Transp. Syst..

[49] Zheng C., Fan X., Wang C., Qi J. (2020). GMAN: A graph multi-attention network for traffic prediction. Proc. AAAI Conf. Artif. Intell..

[50] Zhang X., Huang C., Xu Y., Xia L. (2020). Spatial-temporal convolutional graph attention networks for citywide traffic flow forecasting. Proceedings of the 29th ACM International Conference on Information and Knowledge Managemen, Virtual Event, Ireland, 19–23 October 2020.

[51] Bouchemoukha H., Zennir M.N., Alioua A. (2024). A spatial-temporal graph gated transformer for traffic forecasting. Trans. Emerg. Telecommun. Technol..

[52] Mandlik R., Razavi S., Tighe S. (2025). Traffic speed prediction using MAB-STGNN: Graph neural network built-in model for spatial–temporal graph neural network. Proceedings of the Canadian Society for Civil Engineering Annual Conference 2024.

[53] Edalatpanah S.A., Pourqasem J. (2025). DSTGN-ExpertNet: A deep spatio-temporal graph neural network for high-precision traffic forecasting. Mechatron. Intell. Transp. Syst..

[54] Capone V., Casolaro A., Camastra F. (2025). Spatio-temporal prediction using graph neural networks: A survey. Neurocomputing.

[55] Albalooshi F.A. (2025). Advancing urban planning with deep learning: Intelligent traffic flow prediction and optimization for smart cities. Future Transp..

[56] Abduljabbar R., Dia H., Liyanage S. (2025). Machine learning traffic flow prediction models for smart and sustainable traffic management. Infrastructures.

[57] Gao H., Jiang R., Dong Z., Deng J., Ma Y., Song X. (2024). Spatial-temporal-decoupled masked pre-training for spatiotemporal forecasting. Proceedings of the Thirty-Third International Joint Conference on Artificial Intelligence, Jeju, Korea, 3–9 August 2024.

[58] Tong Z., Liang Y., Sun C., Rosenblum D.S., Lim A. (2020). Directed graph convolutional network. arXiv.

[59] Liu X., Liang Y., Huang C., Zheng Y., Hooi B., Krishnaswamy S. (2023). LargeST: A benchmark dataset for large-scale traffic forecasting. Adv. Neural Inf. Process. Syst..

[60] Pan Z., Ke S., Yang X., Liang Y., Yu Y., Zhang J., Zheng Y. (2021). AutoSTG: Neural architecture search for predictions of spatio-temporal graphs. Proceedings of The Web Conference.

[61] Wu Z., Pan S., Long G., Jiang J., Zhang C. (2019). Graph WaveNet for deep spatial-temporal graph modeling. Proceedings of the 28th International Joint Conference on Artificial Intelligence, Macao, China, 10–16 August 2019.

